# Low levels of intraspecific trait variation in a keystone invertebrate group

**DOI:** 10.1007/s00442-019-04426-9

**Published:** 2019-06-07

**Authors:** Clara A. Gaudard, Mark P. Robertson, Tom R. Bishop

**Affiliations:** 1 Department of Zoology and Entomology, Centre for Invasion Biology, University of Pretoria, Pretoria, 0002 South Africa; 2 Department of Earth, Ocean and Ecological Sciences, University of Liverpool, Liverpool, UK

**Keywords:** Ants, Elevation, Functional traits, Intraspecific, Interspecific, Phylogeny, Southern Africa

## Abstract

The trait-based approach to ecology promises to provide a mechanistic understanding of species distributions and ecosystem functioning. Typically, trait analyses focus on average species trait values and assume that intraspecific variation is small or negligible. Recent work has shown, however, that intraspecific trait variation can often contribute substantially to total trait variation. Whilst many studies have investigated intraspecific variation in plants, very few have done so for invertebrates. There is no research on the level of intraspecific trait variation in ants (Hymenoptera: Formicidae), despite the fact that there is a growing body of literature using ant morphological trait data and demonstrating that these insects play important roles in many ecosystems and food webs. Here, we investigate the intraspecific variability of four commonly used ant morphological traits from 23 species from the Maloti-Drakensberg Mountains of southern Africa. In total, we measured 1145 different individuals and made 6870 trait measurements. Intraspecific variation accounted for only 1–4% of total trait variation for each of the four traits we analysed. We found no links between intraspecific variation, phylogeny and elevation. On average, six individuals generated robust species means but under biased sampling scenarios 20 individuals were needed. The low levels of intraspecific morphological variation that we find suggest that the approach of using mean species traits is valid, in this fauna at least. Regardless, we encourage ant trait ecologists to measure greater numbers of individuals, especially across gradients, to shed further light on intraspecific variation in this functionally important group of insects.

**Electronic supplementary material:**

The online version of this article (10.1007/s00442-019-04426-9) contains supplementary material, which is available to authorized users.

## Introduction

A major goal of ecology is to understand the geographic distribution of biodiversity and its role in maintaining functioning ecosystems. To this end, researchers are increasingly using functional traits, measurable features of individual organisms that influence their fitness (McGill et al. 2006; Violle et al. 2007), to mechanistically explain variation in ecosystem processes (Cadotte 2017; Lyu et al. 2017) and the constraints on biodiversity (Lamanna et al. 2014). Trait-based ecology, however, often fails to incorporate intraspecific variation (Bolnick et al. 2011; Violle et al. 2012). By focussing on species’ trait means, and ignoring within-species trait variation, analyses risk exaggerating interspecific differences and underestimating niche overlap and breadth (Bolnick et al. 2011; Violle et al. 2012). Ultimately, this may lead to researchers drawing false conclusions about the underlying mechanisms shaping biodiversity patterns. As a result, understanding the extent and importance of intraspecific trait variation across a range of taxa and regions has become critical.

Several studies have shown that the inclusion of intraspecific trait variation can influence the interpretation of ecological data. For example, the relative positions of species in multivariate trait ordinations can depend on whether intraspecific variation is included or not (Albert et al. 2010). This could alter the calculation and interpretation of functional diversity metrics (Villéger et al. 2008). Furthermore, Jung et al. (2010) have shown that the detection of community assembly mechanisms in European plants was dependent on whether intraspecific trait data were included. Without intraspecific data, habitat filtering and niche differentiation were more difficult to detect. These results are encouraging ecologists to refocus on the individual organism (Bolnick et al. 2011). A number of studies are now using intraspecific variation to study trait distributions across gradients (Classen et al. 2017; Luo et al. 2016b; Swenson 2011) and at different scales (Luo et al. 2016a; Messier et al. 2010). Additionally, new statistics and modelling approaches have been developed that take advantage of intraspecific trait variation (Carmona et al. 2016; Laughlin et al. 2012; Violle et al. 2012).

Collecting intraspecific trait data from large numbers of individuals, however, can involve high logistical, financial and time costs (Baraloto et al. 2010). In some contexts, such as when using historical collections or working with rare species, it is almost impossible to overcome these costs. As a result, a key goal for many researchers is to better understand intraspecific variation and to assess whether ignoring it is likely to be a problem for downstream analyses in particular contexts and taxa (Albert et al. 2010; Griffiths et al. 2016). Several studies on plants, for example, have shown that intraspecific variation can range from 10% to more the 40% of total trait variation (Albert et al. 2010; Burton et al. 2017; Luo et al. 2016b; Messier et al. 2010; Siefert et al. 2015). These values suggest that ignoring intraspecific variation would be problematic in these cases, especially when the variation is structured across spatial or environmental gradients. In contrast, a recent study on Neotropical dung beetles found very low levels of intraspecific trait variation (Griffiths et al. 2016). Indeed, Griffiths et al. (2016) argue that the failure to incorporate intraspecific data in their context does not lead to a significant loss of information in subsequent analyses.

In this study, we aim to assess the degree of intraspecific morphological trait variation in a keystone invertebrate group, the ants (Hymenoptera: Formicidae), across a large elevational gradient. Ants are abundant on all of Earth’s continents (except Antarctica) and their relatively solid taxonomy, ease of collection (Alonso and Agosti 2000) and functional importance in many ecosystems (Griffiths et al. 2018; Zelikova et al. 2011) makes them a popular study taxon. As a result, there is a large and growing literature of trait-based analyses on worker ants (Arnan et al. 2017; Bishop et al. 2015; Gibb et al. 2014; Silva and Brandão 2010; Weiser and Kaspari 2006). To date, however, there has been no quantitative assessment of how much morphological trait variation there is within the kinds of datasets collected by ant ecologists. This assessment is critical if researchers are to continue to use this taxon to ask important ecological questions.

Specifically, we ask three questions of ant intraspecific variability. (1) *What is the relative amount of ant morphological trait variation that is held at different ecological scales?* In plants, intraspecific variation may account for 40% or more of total trait variation (Messier et al. 2010; Siefert et al. 2015) but in invertebrates this may be much lower (e.g. Griffiths et al. 2016). Furthermore, a number of studies have shown positive, intraspecific trends in ant body size and colour darkness across elevation (Bernadou et al. 2016; Branstetter 2013). We predict that the majority of trait variation will be held at the interspecific scale, but that there is likely to be some spatially structured intraspecific variation that is held between the different elevational sites from which we have sampled. We explore the amount of variation held at the scales of: individual, plot, elevation, species.

(2) *Is ant intraspecific variation linked to either elevation or to phylogeny?* Previous work has shown that different ant clades may show conservatism in morphological syndromes (Weiser and Kaspari 2006) and trophic position (Pfeiffer et al. 2014). Given this, we predict that the amount of intraspecific variation may also be phylogenetically conserved (i.e. that some clades will be more intraspecifically variable than others). There are no studies showing whether the amount of intraspecific variation in ants changes with elevation, but Classen et al. (2017) found that intraspecific variation in African bees tended to decline with increasing elevation; perhaps as a result of increased energetic constraints at high elevations. We also anticipate finding a link between mean intraspecific trait values (i.e. population means) and elevation, as has been shown before (Bernadou et al. 2016; Branstetter 2013), and that there will be a negative relationship between elevation and the amount of intraspecific variation.

(3) *How many worker ants are needed to generate accurate species level trait means?* Most trait-based analyses on ants use a common set of morphological features to characterise the ecology of particular worker castes but the number of individuals that are measured per species is typically low—around 3–10 individuals (Parr et al. 2017; Weiser and Kaspari 2006). Griffiths et al. (2016) suggest that at least 30 individuals are needed for dung beetles. Based on the thorough analysis by Griffiths et al. (2016), and the lack of this kind of information in other invertebrates, we predict that three to 10 measured individuals will be too small to accurately represent species trait means in ants.

## Materials and methods

### Study site

We collected ant specimens from the Sani Pass, a part of the Maloti-Drakensberg Transfrontier Conservation Area of South Africa and Lesotho. We have sampled the Sani Pass for ants biannually since 2006 (Bishop et al. 2014). The sample of ants used in this study was from the ant fauna sampled in 2009 during the wet season (January). The choice to use the year 2009 for this study was arbitrary. The entire gradient is part of the grassland biome of southern Africa (Mucina and Rutherford 2006).

### Data collection

#### Ant sampling

Full sampling details can be found in Bishop et al. (2014). Briefly, we sampled at eight different elevational sites ranging from 900 m a.s.l. to 3000 m a.s.l. with vertical intervals of 300 m. At each site, there were four sampling plots spaced 300 m apart. Each plot contained 10 pitfall traps with a 50% solution of propylene glycol in each. We left traps out for 5 days and nights. The traps were arranged in two rows of five with 10 m spacing between each trap and each row. In the laboratory, specimens were transferred into 70% ethanol and identified to species or morphospecies level. Morphospecies assignments were independent of the four traits analysed here. As a result, if these morphospecies classifications are actually lumping multiple true species together, this would inflate estimates of intraspecific variation. These specimens are part of the collection held in the Department of Zoology and Entomology at the University of Pretoria. We selected a subset of 23 of the 67 species collected during January 2009. These 23 species were chosen based on the criterion that they had 50 or more individuals available to measure. There were 29 species that met this criterion, but due to logistical constraints we only measured a random subset of 23 species (Appendix S7.4).

The species chosen have a range of ecological strategies and life histories (Fisher and Bolton 2016). For example, *Crematogaster, Monomorium, Pheidole, Tetramorium* and *Solenopsis* are cosmopolitan genera that fill a variety of ant niches. *Crematogaster* are often arboreal specialists, though not in this grassland environment. *Camponotus* is a cosmopolitan genus that is often found at the “herbivorous” end of the ant dietary spectrum (Pfeiffer et al. 2014) and may be the most speciose ant genus globally. *Carebara* is pantropical with minute and cryptic workers. *Streblognathus*, a genus endemic to South Africa and Lesotho, is large and predatory. *Leptogenys* varies in size, is pantropical in distribution but is also characterised as predacious.

This sampling design and specimen collection are typical of those commonly used in ant trait-based ecology (Del Toro et al. 2015; Gibb et al. 2017b; Salas-Lopez et al. 2018).

#### Trait measurements

We measured 50 individuals per species and consider trait means calculated from this number of individuals to represent the true population mean for our study site. We chose 50 since this appears to be a large enough sample size to accurately quantify species trait means according to the recommendations of Griffiths et al. (2016) for dung beetles. The specimens we measured were selected as evenly as possible from across all the sampling plots (four per elevation) and elevational sites (maximum of eight) from which a given species was sampled. This even sampling of specimens ensured that we maximised the number of colonies that we were sampling for each species. One species was represented by a single colony. The maximum number of colonies sampled for a species was 18 and the mean was 9.3. We consider workers found in different plots and from different sites to be from different colonies, as plots at the same elevation were separated by a distance of at least 300 m and different elevations by 300 vertical metres. There are also likely to be different colonies of the same species present within a single plot. We cannot infer this level of detail from our data, however, and so the number of colonies per species that we have sampled is likely higher than our minimum estimates (e.g. 4 plots × 8 sites = 32 known colonies at minimum, if a species is present at all plots). Indeed, even if we had trap-level data it would be impossible to know how many colonies of a species had been caught in a single plot. As a result, we reasonably assume that species caught in different plots represent different colonies and accept that this is probably an underestimate of colony number.

**Fig. 1:**
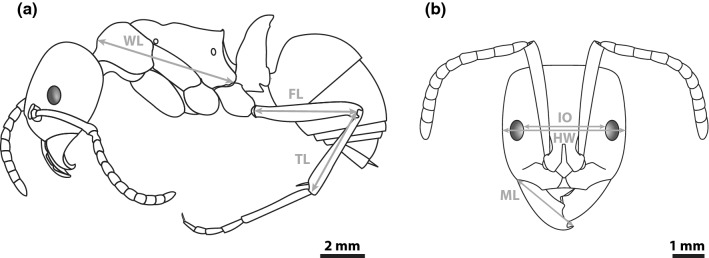
Illustration of the six raw trait measures on a schematic of *Streblognathus peetersi,* one of our measured species. This genus is endemic to South Africa and Lesotho. **a** Traits measured from profile view and **b** traits measured in full-face view. *WL* Weber’s length, *FL* hind femur length, *TL* hind tibia length, *IO* interocular distance, *HW* head width, *ML* mandible length. Eye position was calculated by subtracting interocular distance from head width. Leg length was calculated by summing hind femur and hind tibia lengths

We measured four worker morphological traits that are regularly used in the literature. Two of these were compound traits made up from multiple raw traits. In total, we measured six raw traits (Fig. 1). The four compound traits were: (1) Weber’s length, (2) eye position, (3) leg length, and (4) mandible length. Weber’s length represents body size and was measured from the base of anterior slope of pronotum to the lower posterior angle of propodeum (Brown 1953). Eye position was calculated by subtracting interocular distance from total head width across the eyes. The larger the value, the more dorsally positioned the eyes are. Zero values were assigned for species with eyes that were too small to measure. Leg length was calculated as the sum of the length of the hind tibia and femur. Mandible length was measured from insertion to tip. These traits are not relative to body size.

We only measured traits from the minor worker caste in each species, where applicable. When polymorphic, we measured only the most common worker size—typically the smallest. Consequently, this study does not incorporate issues of caste identity but focuses on the most common worker ants. This is routinely done in ant functional trait ecology (Parr et al. 2017). In addition, major and solider castes are not common in our pitfall traps, which are biased toward actively foraging workers.

We measured traits using an ocular micrometer attached to a Stemi 2000 stereomicroscope (Carl Zeiss Microscopy, Jena, Germany), as well as an M80 stereomicroscope (Leica, Solms, Germany). We used the highest magnification available which would allow the structure being measured to fit within the range of the ocular micrometer. All measurements were recorded to the nearest 0.01 mm.

The four traits that we have measured are commonly used in the ant trait literature and are thought to link to ant diet preference and habitat use (Parr et al. 2017). Weber’s length is used as a proxy for overall size which relates to a number of metabolic and dietary characteristics (Parr et al. 2017; Traniello 1987). Eye position and leg length can indicate the kinds of habitat that are used by ant species (Gibb and Parr 2010). Eye position and mandible length are expected to be linked to resource use and predatory specialisation (Fowler et al. 1991; Weiser and Kaspari 2006).

We tested the repeatability of the trait measurements by measuring the head width of three specimens from two different species repeatedly over 15 separate days. The average standard error of these six specimens was calculated as an average recorder error per specimen.

### Statistical analysis

We used R version 3.3.2 (R Core Team 2017) for all statistical analyses and data manipulations.

#### What is the relative amount of ant functional trait variation that is held at different ecological scales?

To understand the degree and source of variability in ant traits, we used a variance component analysis (Messier et al. 2010). First, the data were normalised by log_10_ transforming each of the four traits. Second, linear mixed-effects models (lme) from the ‘nlme’ package (Pinheiro et al. 2017) were fitted for each trait. Individual worker ants were nested within plot, within elevation, within species and included as a random effect. This structure allows us to determine how much trait variability is held between individuals within a plot, between different plots in the same elevation, between different elevations and between different species. Pitfall traps were not included in the random effects structure as samples were pooled at the plot level. We do not include any fixed effects; therefore, we do not report the traditional mixed-effects model outputs. Third, we used a variance component analysis (varcomp), from the ‘ape’ package (Paradis et al. 2004), on each of the mixed-effects models. We estimated the 95% confidence intervals around the variance components by bootstrapping the data 1000 times with replacement using only 70% of the original number of specimens. We also visually assessed intraspecific variation by calculating the coefficient of variation (CV) per trait per species, where CV is defined as the standard deviation divided by the mean and expressed as a percentage.

#### Is ant intraspecific variation linked to either elevation or to phylogeny?

We linked both intraspecific variance and intraspecific means to elevation. First, we calculated the CV and mean of each trait at each elevation for each species. We limited this to elevations that had more than one individual of a given species. Furthermore, only species which occurred at four or more elevations were considered (10 species). For each of these species and each of their traits, a linear regression was used to model the relationship between intraspecific variation (as measured by CV) or intraspecific mean and elevation. We weighted the regression models by the number of individuals measured at each elevation to control for the fact that the number of specimens available at different elevations for each species varies. We put more weight in estimates of CV and the mean made from elevations where more individual specimens were measured. For every species and trait, we extracted the slopes (change in CV or mean trait per metre of elevation) and significance values from the regression models. These slopes and significance values were visually inspected to assess any elevational trends for each trait.

The phylogenetic signal of intraspecific variation (as measured by CV) was estimated using an adapted version of the Moreau and Bell (2013) genus-level, time-calibrated ant phylogeny, Pagel’s lambda (Pagel 1999) and Blomberg’s K (Blomberg et al. 2003). This phylogeny did not include *Lepisiota* or *Streblognathus*. They were inserted next to their most closely related sister genera. *Streblognathus* was inserted as sister to *Leptogenys* and *Odontoponera*, and *Lepisiota* as sister to *Prenolepis* and *Plagiolepis*. We averaged the CV of each trait and species to the genus level and used the ‘phytools’ package (Revell 2012) to run likelihood ratio tests to determine whether Pagel’s lambda or Blomberg’s K for each trait departed significantly from the null expectation of no phylogenetic signal.

#### How many worker ants are needed to generate accurate species level means?

We assessed how many individuals were needed to produce accurate species level means using a resampling procedure. First, we resampled with replacement from between 2 and 50 individuals for every species. We chose this large range so that we could investigate what happened to the estimated mean using either very few individuals (2, likely to be inaccurate) or very many (50, likely to be accurate). We resampled 500 times for each number of individuals (2–50). Second, we calculated the accuracy of resampled means compared to the population mean as:

$$ {\text{Accuracy}} = 100 - \left( {\frac{{\left| {\bar{x} - \mu } \right|}}{\mu }} \right) \times 100 $$

where $\bar{x}$ is a resampled mean and $\mu$ is the population mean calculated from the original data. This measure of accuracy records the absolute difference between resampled means and the population mean, expressed as a percentage of the population mean. Values closer to 100 indicate that a resampled mean is closer to the population mean. This gives a value of accuracy for each resample. Third, we averaged the accuracy scores across resamples within a given number of individuals for each species and each trait to represent a “most likely” scenario. We also averaged the accuracy across the worst performing 50 resamples for each species and each trait to represent a “worst case” scenario. We then plotted accuracy as a function of the number of individuals for each species for both the “most likely” and “worst case” scenarios. Finally, for each scenario, we calculated the threshold number of individuals that were needed to achieve an accuracy of 95% for either 90% of the species or for 50% of the species.

## Results

We measured 50 individuals for each of 20 species. For the remaining three species, we measured 51, 49 and 45 individuals; therefore, a total of 1145 individuals were measured and ~ 6870 trait measurements were taken. The coefficient of variation for repeated measures was on average 0.9% (Appendix S1), demonstrating that recorder error of these measurements was low. The largest ants were *Streblognathus peetersi* and the smallest were from a species in the genus *Carebara*. They had maximum and minimum Weber’s lengths of 5.64 mm and 0.35 mm, respectively. This range is representative of the size variation present in ants from this area (Bishop et al. 2015).

### What is the relative amount of ant functional trait variation that is held at different ecological scales?

**Fig. 2:**
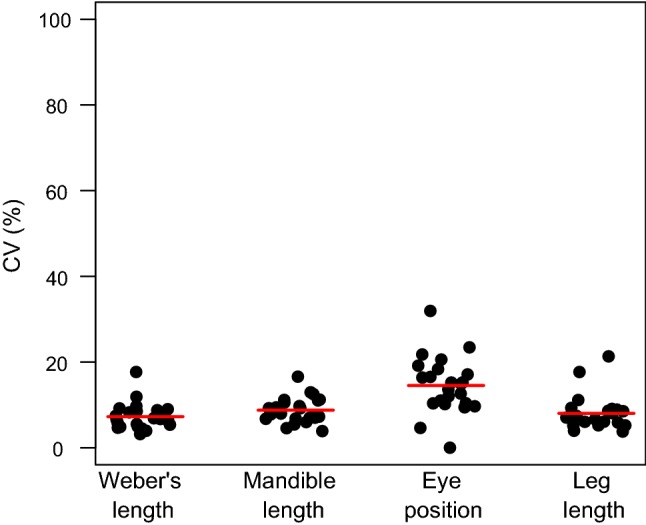
Coefficient of variation (CV) for each species and trait. Each point represents the CV of a single species for a single trait. Points were jittered in the *x* axis to increased visibility of points. Red lines indicate the mean CV for each trait

Interspecific variance made the largest contribution to morphological trait variability for all four traits. Interspecific variance accounted for between 96 and 98% of the partitioned variance, whereas intraspecific variance accounted for only one to four percent of total variability (Table 1). There was almost no variation held between elevations within species (0–2%, Table 1), or between plots of a given elevation (0.12–1.16%, Table 1). There was a larger fraction held between individuals of a species within plots (0.9–2.66%, Table 1). The average coefficient of variation (CV, Fig. 2) varied from between 7 and 9% for Weber’s length, mandible length and leg length and was 14.5% for eye position.

**Table 1:** Results of variance partitioning for each trait (*n* = 1145 ant specimens)

	Interspecific	Intraspecific		
	Between species	Between elevations	Between plots	Between individuals (+ error)
Weber’s length	97.5 (96.94–97.78)	0.81 (0.33–1.31)	0.26 (0.35–1.1)	1.44 (0.94–1.41)
Mandible length	96.82 (96.12–97.21)	0.95 (0.32–1.47)	0.13 (0.35–1.16)	2.1 (1.44–1.99)
Eye position	96.79 (96.15–97.2)	0.41 (0–0.76)	0.12 (0.34–1.13)	2.68 (1.89–2.66)
Leg length	97.35 (96.66–97.81)	1.29 (0.57–1.97)	0.22 (0.29–1)	1.14 (0.73–1.09)

Cells contain the percentage of total variation held at each scale. The parentheses show the 2.5% and 97.5% percentiles of the variance estimates, and were calculated by bootstrapping (1000 runs with 802 randomly sampled specimens with replacement)

### Is ant intraspecific variation linked to either elevation or to phylogeny?

**Fig. 3:**
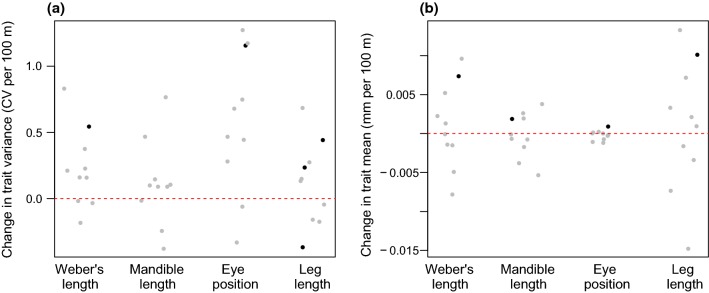
The change in trait variance (**a**) and trait mean (**b**) per 100 m of elevation. Each data point represents the slope value extracted from a linear regression of CV or trait mean against elevation for each species. Grey dots are not significant (linear regression, *p* > 0.05). Black dots are significant (linear regression, *p* < 0.05). The dotted red lines mark a slope value of 0. Positive values mean that the variance or the mean increases with increasing elevation; negative values indicate that they decrease with increasing elevation. Slope values are expressed in change per 100 m as an arbitrary choice to improve readability and interpretation of the *y* axis

Overall, we found weak evidence for changes in intraspecific mean trait values or intraspecific variation across elevation. Out of 40 species-by-trait combinations (4 traits for 10 species), only five showed significant relationships between intraspecific variation and elevation (Fig. 3a, Appendix S2). *Pheidole UN01* had a positive relationship between elevation and variance in Weber’s length and leg length. *Crematogaster natalensis* and *Tetramorium frigidum* had greater variance in eye position and leg length, respectively, at higher elevations. *Monomorium UN01* had greater variation in leg length at lower elevations. Out of 40 species by trait combinations, four showed significant relationships between mean trait values and elevation. *Tetramorium bothae* displayed a significant positive relationship between each trait and elevation (Fig. 3b, Appendix S2).

The amount of intraspecific variation (CV) was not significantly conserved across phylogeny for any of the four traits. For each trait, both Pagel’s *λ* and Bloomberg *K* tests were not significant (*p* > 0.05, Appendix S2). This result implies that closely related genera do not have more similar proportions of intraspecific variation than would be expected by chance.

### How many worker ants are needed to generate accurate species level means?

**Fig. 4:**
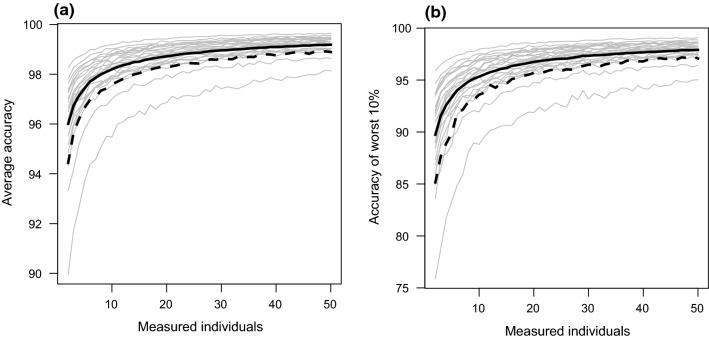
Plots showing the changing accuracy of resampled means with different number of individuals for Weber’s length. Grey trace lines represent changes in accuracy for individual species. Thick black solid lines indicate the median accuracy (50% of the fauna). Thick black dashed lines indicate the 10th percentile accuracy (encompassing 90% of the fauna). **a** The most likely scenario (average accuracy across all resamples). **b** The worst case scenario (average accuracy using only the worst 10% of the resamples)

As expected, the accuracy of resampled trait means increased with the increasing number of individuals sampled (Fig. 4, Appendix S3). Understandably, more individuals were needed to achieve our accuracy threshold of 95% when considering the worst case scenario (average accuracy of the worst performing 10% of resamples) and 90% of the fauna (Table 2). The fewest number of individuals is needed to reach our accuracy threshold when considering the most likely scenario (average accuracy of all 500 resamples) for only 50% of the fauna (Table 2). Across all traits, and considering 90% of the fauna, six individuals per species were needed to generate accurate species level means for the most likely scenario. This number rose to 20 individuals under the worst case scenario.

**Table 2:** Number of individuals needed to achieve greater than 95% accuracy for either 50% or 90% of the fauna when considering either the average of all resamples (most likely scenario) or the average of the worst 10% of resamples (worst case scenario)

Trait	Most likely scenario		Worst case scenario	
	50% of fauna	90% of fauna	50% of fauna	90% of fauna
Weber’s length	2	3	8	16
Mandible length	2	5	12	25
Eye position	6	13	34	NA
Leg length	2	4	9	19

The variability of eye position was such that more than 50 individuals were required to achieve threshold accuracy for 90% of the fauna under the worst case scenario

## Discussion

Our study builds on previous work quantifying the intraspecific variability of traits. Little is known about intraspecific variability in invertebrates (but see Classen et al. 2017; Griffiths et al. 2016; Ohkawara et al. 2017), and even less about its importance in ants. Our results show that the total variation of ant traits from our study site is almost entirely accounted for by interspecific variation (96–98%), not intraspecific variation (1–4%, Table 1). This is in contrast to many of the studies on plants that have reported substantial amounts (10–40%) of intraspecific variation (Albert et al. 2010; Jung et al. 2010; Messier et al. 2010). There are potentially three reasons for this discrepancy.

The first concerns different spatial and environmental scales. Albert et al. (2011) proposed the spatial variance partitioning (SVP) hypothesis to understand how the relative amounts of intra- and interspecific variation change with spatial and environmental scale. As scale increases, more environmental variation is captured. As this happens, intraspecific variation saturates as entire species’ niches and ranges are captured by the scope of the study. Interspecific variation continues to increase past this point, however, as new species are encountered (Albert et al. 2011). Our study system in the Maloti-Drakensberg Mountains (900–3000 m a.s.l.) encompasses a large fraction of the temperatures and conditions that ants living in the grassland biome of southern Africa will experience. Indeed, we have already shown a pattern of species turnover in ants across this elevational gradient, confirming that beta diversity increases with scale and likely contributes to the balance between interspecific and intraspecific variation (Bishop et al. 2015). Consequently, our data are likely to be representative of a “large-scale” study system as explained by the SVP hypothesis and, therefore, are expected to have a higher proportion of interspecific trait variation. Our scale of study, however, is common in the trait-based ecology literature for both animals and plants and we argue that our sampling design and dataset is representative of many community and macroecological datasets (Del Toro et al. 2015; Lamanna et al. 2014; Swenson et al. 2011).

The second reason for the low levels of intraspecific variation that we find in ants may be due to a lack of phenotypic plasticity. Plants are often reported to have high levels of phenotypic plasticity and, consequently, intraspecific variation (Auger and Shipley 2013; Kazakou et al. 2014; Rozendaal et al. 2006). This is often explained by their sessile nature (Pigliucci 2005; Van Kleunen and Fischer 2005). Plants must be able to adapt to conditions in a plastic way, by altering their morphology or development, rather than simply moving themselves to more favourable environments as animals do. Whilst entire ant colonies may behave plastically by changing the ratios of sexual to non-sexual brood (Sundstrom et al. 1996), or altering foraging trails (Kost et al. 2005), individual ant workers are often raised within a controlled environment inside the nest and once adult workers emerge their morphology is fixed (Hölldobler and Wilson 1990). As a result, we may expect there to be much lower levels of trait plasticity in ant morphology at the level of the individual worker in comparison to plants. Our results indirectly support this view but further experimental work is needed to fully understand whether the development of ant worker morphology can change plastically in response to the environment.

The third reason is that we only measure workers from a single caste. This is a common strategy in ant functional ecology (e.g. Salas-Lopez et al. 2018; Weiser and Kaspari 2006). This is a practical compromise when faced with dimorphic or polymorphic worker ants. Some of the species we sample here show worker di- or polymorphism to varying degrees (*Pheidole, Carebara, Monomorium, Solenopsis* and *Camponotus*). Clearly, incorporating measurements from both minor *Pheidole* workers and their large-headed major counterparts would greatly increase the intraspecific variation that we find for *Pheidole* species. It is unclear, however, if any trait-based study on ants would lump polymorphic workers together like this, and not analyse them as effectively “separate species”. In consequence, our data can only show that the minor caste shows little intraspecific variation. Whether this conclusion holds for the diversity of ant worker castes in different phylogenetic groups and geographic contexts is an unexplored question.

Of the four traits we analyse, eye position had the largest proportion of intraspecific variation (3.2%, Table 1) and the highest average coefficient of variation (Fig. 2). Since this trait is made up of a combination of two measured traits, there is potentially double the amount of recorder error. Eye position was also the smallest measured trait, making it the most difficult to measure. Head width (which is used to calculate eye position), however, shows high repeatability and low recorder error (Appendix S1), and none of the traits we measure have a relationship between intraspecific variation and absolute size (Appendix S4). Consequently, it is unlikely that explanations of recorder or measurement error explain the higher intraspecific variability we see in this trait. Rather, our data suggest that eye position really does have more intraspecific variation than the other traits, even if the magnitude of this variability is still relatively small and not often exceeding a CV of 20%.

Previous work on ants has shown Bergmannian clines where worker body size is larger at higher elevations and latitudes at the intraspecific level (Bernadou et al. 2016; Branstetter 2013; Heinze et al. 2003). These findings come from one European species, *Leptothroax acervorum*, and from Neotropical *Stenamma*. We find only one case of increased worker size at high elevations (Fig. 3b), *Tetramorium bothae*. Furthermore, our variance partitioning analysis revealed that the amount of intraspecific variation associated with elevational differences is typically less than that found between individuals within the same plot (“between elevations” vs. “between individuals”, Table 1). In our data, the little intraspecific variation that we detected can largely be found within a single sampling plot, not structured across the elevational gradient. Notably, Nowrouzi et al. (2018) also found limited evidence for intraspecific body size changes across elevation for ants, in the Australian Wet Tropics. Combined, these findings suggest that clines in ant intraspecific morphological trait means are the exception, rather than the rule.

We found very few links between the amount of intraspecific variation present and elevation (Fig. 3a). This is in contrast to Classen et al. (2017) who found that, on average, African bees were less intraspecifically variable at high elevations. Classen et al. (2017) suggested that this pattern may be caused by energetic constraints imposed, in part, by the reduced available area at high elevation on Mt. Kilimanjaro. The reasoning is that smaller available areas provide less energy to be exploited by organisms and that these energetic constraints prevent a wide range of phenotypes (i.e. intraspecific variability) from existing. The Maloti-Drakensberg, that we sample, however, does not show such a strong negative relationship between available area and elevation that Mt. Kilimanjaro does. Rather, available area in the highest elevations is relatively constant and actually increases at the top of the Drakensberg escarpment (Appendix S5). This lack of the same area and energy constraints in our study system may explain why we do not see a strong signal of decreased intraspecific variance at high elevations.

Our data also provide no evidence for phylogenetic conservatism in intraspecific variation. This is perhaps not surprising, given that intraspecific variation itself was so low. It does contrast with widespread, accepted knowledge amongst ant ecologists that subfamilies, and sometimes genera, are often conserved in their morphology (Weiser and Kaspari 2006), trophic positions (Pfeiffer et al. 2014) and other life history characters such as queen number and colony founding strategy (Hölldobler and Wilson 1990).

Our resampling analysis shows that, on average, measuring around six individual worker ants is enough to generate a trait mean close to the population mean (Most likely scenario: Table 2). When we considered a worst case scenario, where extreme combinations of individuals were sampled and more extreme trait means were estimated, we needed many more individuals to bring the estimated trait means within 5% of the population means (Worst case scenario: Table 2). This result is important because many studies and protocols in ant trait ecology measure relatively few individuals: from three to 10 (Bishop et al. 2015; Gibb et al. 2014; Parr et al. 2017; Silva and Brandao 2014; Weiser and Kaspari 2006). The results here suggest that that measuring six individuals is not likely to seriously skew the estimated trait means of ants, however, we recommend that ant ecologists err on the side of caution and take trait data from 20 individuals. This will ensure that species level trait means are close to that estimated using 50 individuals, regardless of extreme sampling bias. This will also provide opportunity to gather further quantitative data on intraspecific variation in ants, particularly across environmental gradients.

A final point of consideration is whether our results can be generalised to species pools of different sizes or geographic contexts. We compared our dataset to a global database on ant species abundances (Gibb et al. 2017a) and present full details in Appendix S7. In short, we find that the full species pool of our site is typical of studies globally, and that it has higher than average phylogenetic diversity. Furthermore, our subset of 23 species has average phylogenetic diversity and above average morphological diversity compared to our wider species pool. Given these points, and the data currently available, we see no cogent reason why our findings will not be extended to a variety of different ant faunas. Regardless, the only way to tell is to collect more data on intraspecific variation in ants, and we encourage other researchers to do so.

In conclusion, we have performed the first assessment of intraspecific variation in ants. In general, intraspecific variation is very low and is not strongly linked to changes in elevation, and is not phylogenetically conserved. We find that, on average, very low numbers of individual ant workers are needed to accurately capture population trait means. Regardless, the accuracy of estimated means can differ from population means substantially in extreme cases and we recommend collecting ant morphological trait data from around 20 individuals to avoid this potential pitfall.

## Electronic supplementary material

Below is the link to the electronic supplementary material.

Supplementary material 1 (DOCX 7133 kb)

## Data Availability

Raw data, analysed data (e.g. resampling repeats) and R code will be archived in the Dryad Digital Repository upon publication.
