# Supplementary material for: Low levels of intraspecific trait variation in a keystone invertebrate group
Source: Oecologia. 2019 Jun 7;190(4):725–35. doi: 10.1007/s00442-019-04426-9 (PMC6704090; doi:10.1007/s00442-019-04426-9)
Supplement: Supplementary file 1 — Supplementary material 1 (DOCX 7133 kb) [file 442_2019_4426_MOESM1_ESM.docx]

**Low levels of intraspecific trait variation in a keystone invertebrate group - Appendices**

Clara A. Gaudard, Mark P. Robertson and Tom R. Bishop

**Appendix S1: Repeatability of measurements**

We repeatedly measured the head width of six specimens from two species over two months. All measurements were made by Clara Gaudard, who also took all the measurements for the main data set. We calculated the coefficient of variation of these repeat measurements per specimen and these are presented in Table S1.

| **Table S1.1.** Repeatability of head width measurements on six specimens. The average of the repeated measures is given, along with the standard deviation and the coefficient of variation expressed as a percentage. | | | | | |
| --- | --- | --- | --- | --- | --- |
| Species | Individual ID code | Repeated measures | Average (mm) | Standard deviation (mm) | CV (%) |
| *Streblognathus peetersi* | INTRA-0002 | 15 | 3.78 | 0.04 | 0.93 |
| *Streblognathus peetersi* | INTRA-0015 | 15 | 3.73 | 0.03 | 0.76 |
| *Streblognathus peetersi* | INTRA-0040 | 15 | 3.64 | 0.03 | 0.83 |
| *Crematogaster UN02* | INTRA-0119 | 15 | 1.21 | 0.02 | 1.28 |
| *Crematogaster UN02* | INTRA-0132 | 15 | 1.17 | 0.01 | 1.21 |
| *Crematogaster UN02* | INTRA-0210 | 15 | 1.04 | 0.01 | 0.61 |

**Appendix S2: Environmental and phylogenetic links**

Additional figures and tables showing the relationship between intraspecific variation, the environment and phylogeny.


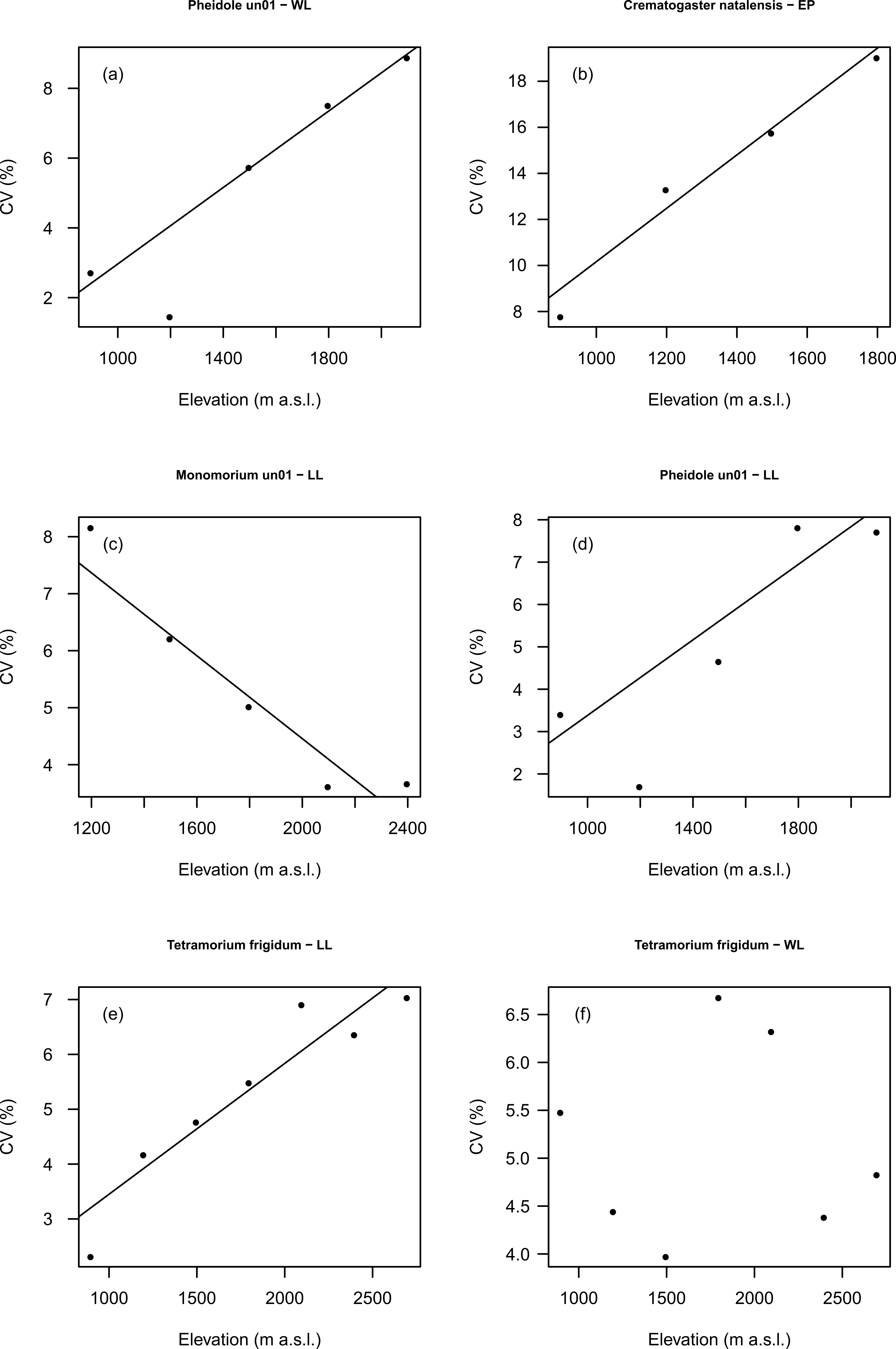


**Figure S2.1.** Relationship between coefficient of variation (CV) and elevation for the 5 species/trait combinations that showed significant relationships (a-e) and another combination that was not significant (f).


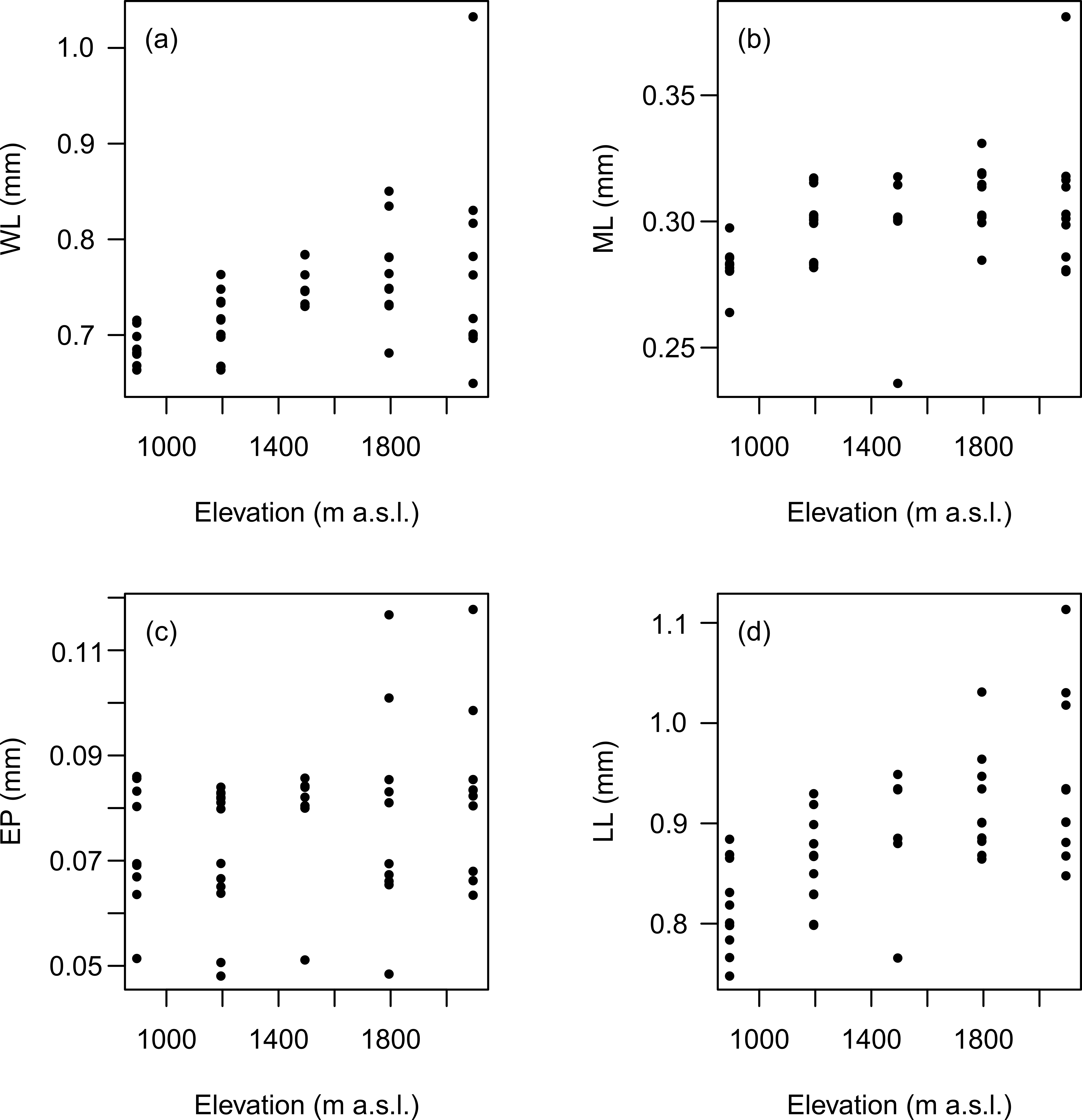


**Figure S2.2.** Relationship between trait measures and elevation for *Tetramorium bothae*. All relationships are significant (linear regression, p < 0.05). Traits get larger with increased elevation.

| **Table S2.1.** Phylogenetic signal of intraspecific variation for each trait. No significant phylogenetic conservatism was detected. | | | | |
| --- | --- | --- | --- | --- |
|  |  |  |  |  |
| Trait | Pagel’s λ | P value | Blomberg’s K | P value |
|  |  |  |  |  |
| Weber's length | 7.98E-05 | 1 | 0.81 | 0.23 |
| Mandible length | 7.98E-05 | 1 | 0.6 | 0.69 |
| Eye position | 7.98E-05 | 1 | 0.55 | 0.74 |
| Leg length | 7.98E-05 | 1 | 0.68 | 0.56 |

**
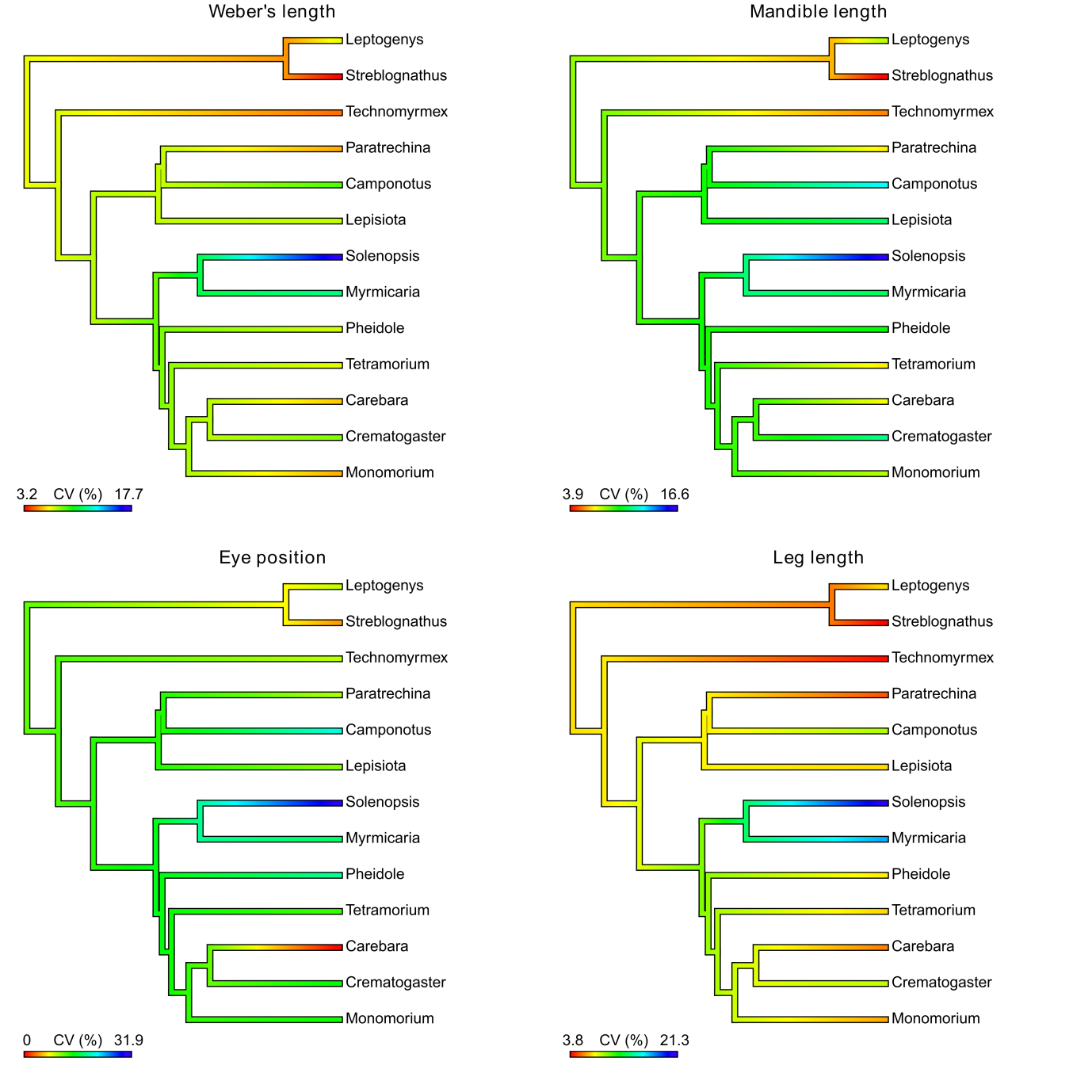
**

**Figure S2.2.** Genus level phylogenies for each trait visually showing the amount of intraspecific variation (CV, rainbow colours). Amount of CV on internal nodes and branches estimated using maximum likelihood estimation of ancestral states.

**Appendix S3: Accuracy of resampled data**


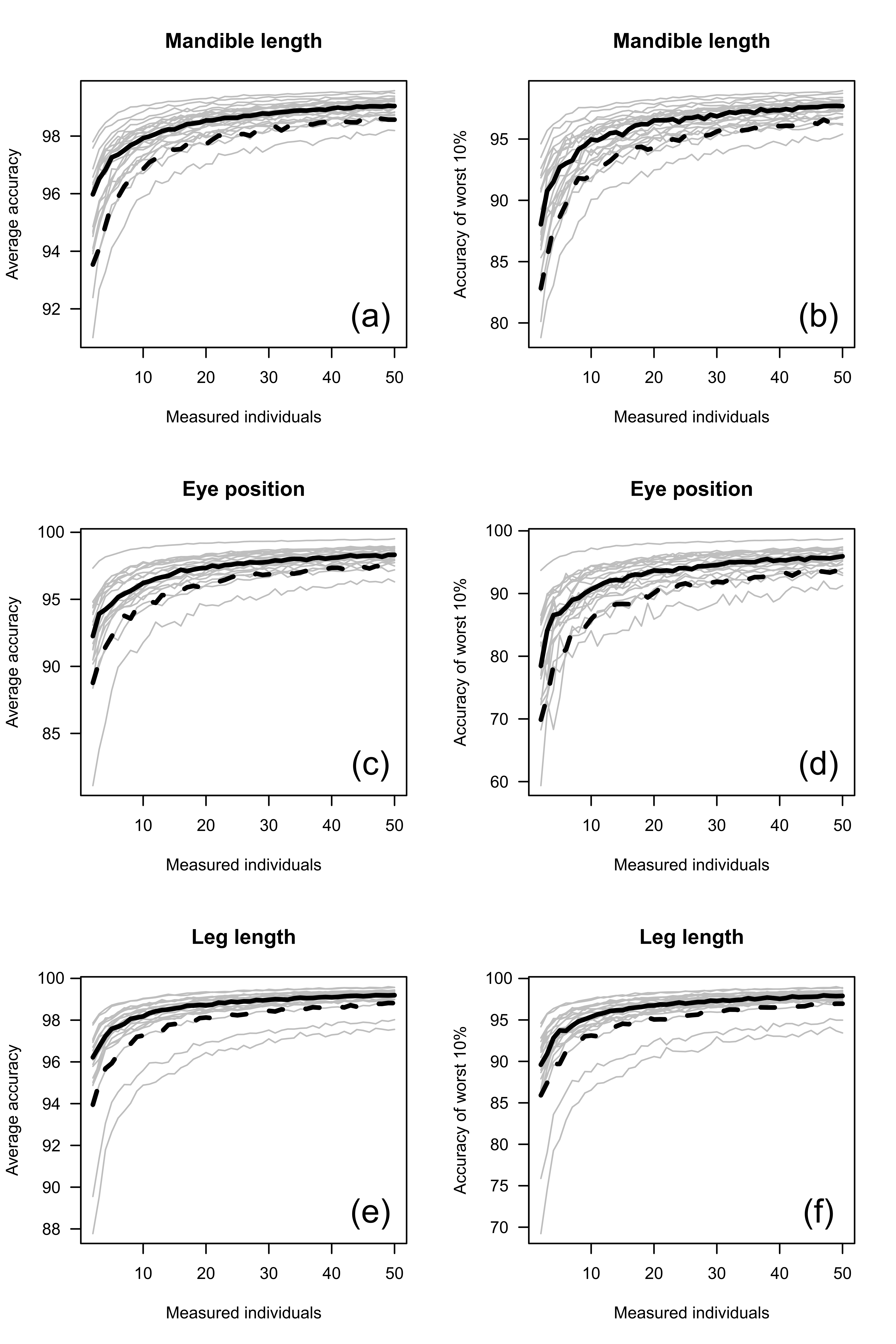


**Figure S3.1.** Accuracy of resampled mandible length, eye position and leg length using either the average accuracy of all resamples (a, c, e) or the average accuracy of the worst 10% (b, d, f). Grey trace lines represent individual species. Thick black lines indicate the average taken across species.

**Appendix S4: Absolute size and intraspecific variation**


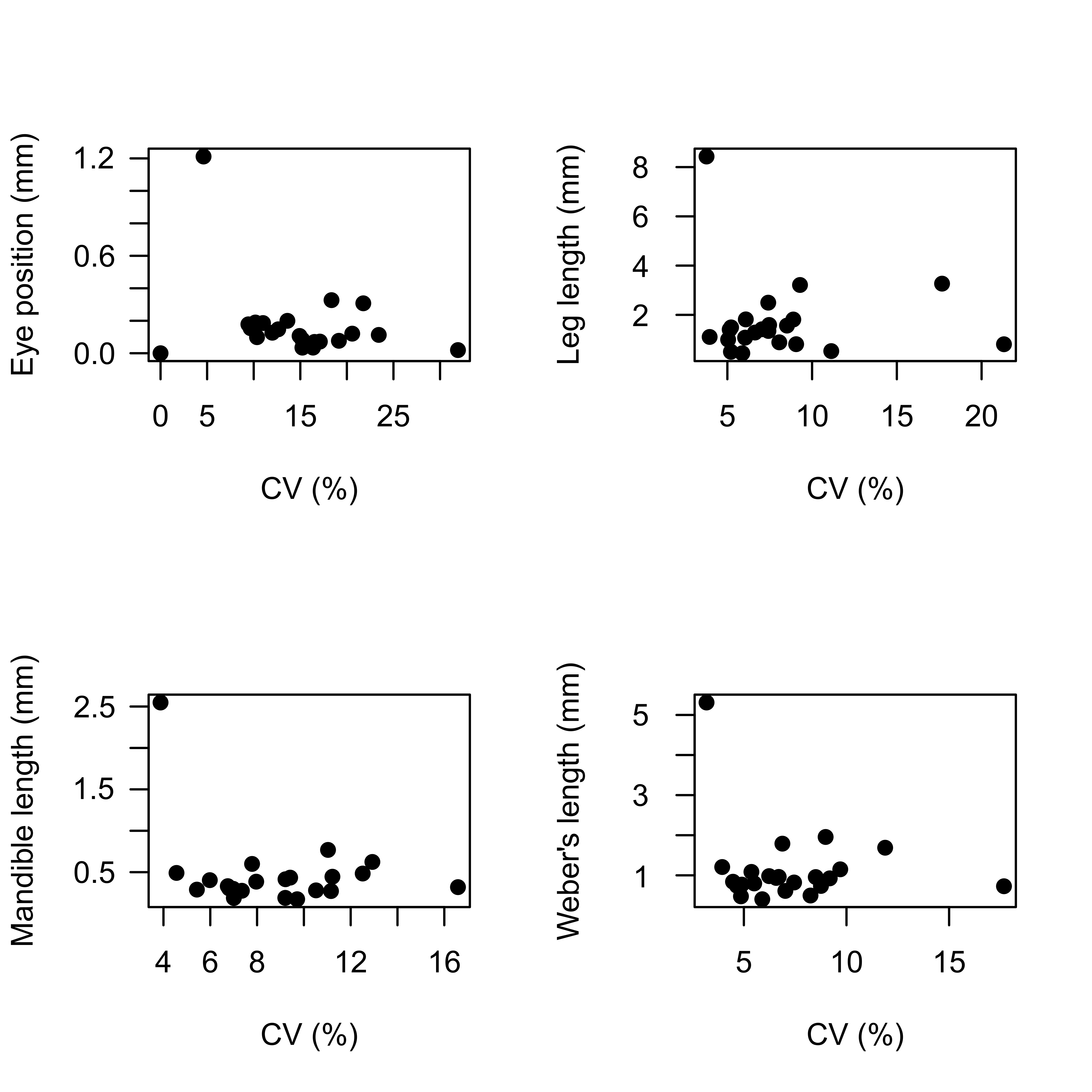


**Figure S4.1.** Relationship between measured average trait values and intraspecific variation (coefficient of variation, CV). Each data point represents a species. CV (x-axis) was calculated using all specimens for a given species, for a given trait. Traits (y-axis) were calculated using all specimens of a given species and are presented as the population average.

**Appendix S5.** **Available area across elevation**


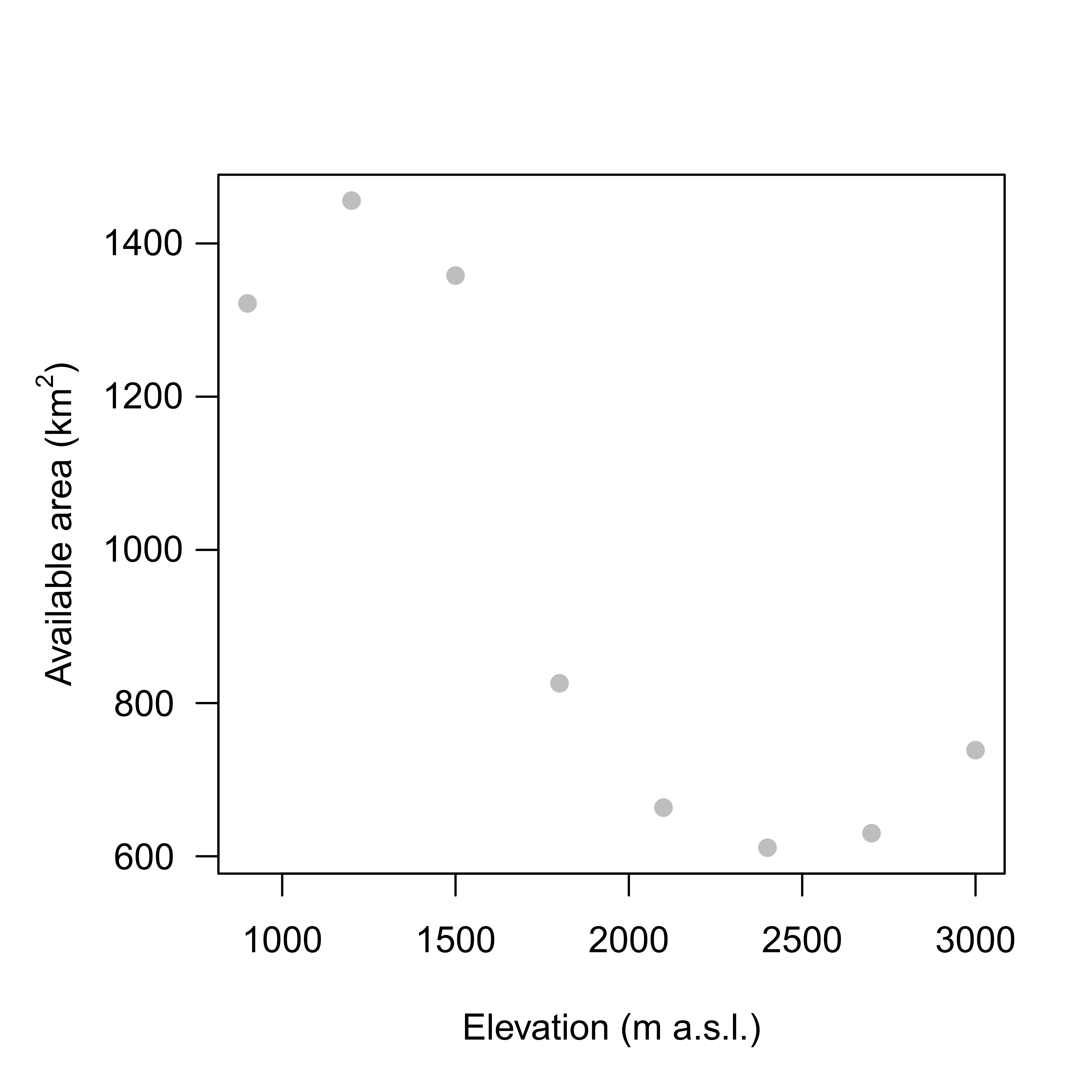


**Figure S5.1**. Plot showing how available area changes with elevation. Available area calculated using a digital elevation model. Area for each elevation (± 100 vertical metres) was extracted from a 100 km radius around our sampling sites.

**Appendix S6.** **Species list and record of elevational sampling.**

| Table S5.1. Table showing number of specimens for each species from each plot and elevational band. | | | | | | | | | | | | | | | | | | | | | | | | | | | | | | | | |
| --- | --- | --- | --- | --- | --- | --- | --- | --- | --- | --- | --- | --- | --- | --- | --- | --- | --- | --- | --- | --- | --- | --- | --- | --- | --- | --- | --- | --- | --- | --- | --- | --- |
|  | Elevation (m a.s.l.) and plot ID | | | | | | | | | | | | | | | | | | | | | | | | | | | | | | | |
|  | 3000 | | | | 2700 | | | | 2400 | | | | 2100 | | | | 1800 | | | | 1500 | | | | 1200 | | | | 900 | | | |
| Species | 1a | 1b | 1c | 1d | 2a | 2b | 2c | 2d | 3a | 3b | 3c | 3d | 4a | 4b | 4c | 4d | 5a | 5b | 5c | 5d | 6a | 6b | 6c | 6d | 7a | 7b | 7c | 7d | 8a | 8b | 8c | 8d |
| *Camponotus* sani01 | 1 |  | 20 | 4 |  |  |  | 21 |  |  | 4 |  |  |  |  |  |  |  |  |  |  |  |  |  |  |  |  |  |  |  |  |  |
| Carebara sani01 |  |  |  |  |  |  |  |  |  |  |  |  |  |  |  |  | 1 | 2 | 2 |  | 15 | 7 | 8 | 5 |  |  |  |  |  |  | 5 |  |
| Crematogaster natalensis |  |  |  |  |  |  |  |  |  |  |  |  |  | 1 |  |  |  |  | 6 |  | 9 | 9 |  |  | 4 | 7 | 3 | 4 |  |  | 7 |  |
| Crematogaster un02 |  |  |  |  |  |  |  |  |  |  |  |  |  |  |  |  | 8 | 8 | 3 | 6 | 3 | 22 |  |  |  |  |  |  |  |  |  |  |
| Lepisiota sani01 |  |  |  |  |  |  |  |  | 2 | 2 | 4 | 4 | 4 | 4 | 2 | 2 | 3 | 3 |  | 1 | 3 |  |  |  | 2 | 2 | 2 | 2 |  |  | 4 | 4 |
| Leptogenys intermedia |  |  |  |  |  |  |  |  | 1 |  | 9 |  |  | 7 |  | 16 |  |  | 1 | 5 |  |  |  | 7 | 2 |  | 2 |  |  |  |  |  |
| Monomorium sani01 | 11 | 19 | 1 | 11 |  | 2 | 5 | 1 |  |  |  |  |  |  |  |  |  |  |  |  |  |  |  |  |  |  |  |  |  |  |  |  |
| Monomorium sani03 |  |  |  |  | 50 |  |  |  |  |  |  |  |  |  |  |  |  |  |  |  |  |  |  |  |  |  |  |  |  |  |  |  |
| Monomorium musicum |  |  |  |  | 1 | 4 |  |  | 11 | 6 | 10 | 11 |  |  | 2 |  |  | 1 |  | 4 |  |  |  |  |  |  |  |  |  |  |  |  |
| Monomorium taedium |  |  |  |  |  |  |  |  |  |  |  |  |  | 2 |  | 8 | 3 | 4 | 3 |  | 2 | 4 | 2 | 2 | 2 | 2 | 5 | 1 |  | 3 | 6 | 1 |
| Monomorium un01 |  |  |  |  |  |  |  |  | 8 |  | 2 |  | 8 | 8 |  |  |  | 3 | 4 | 3 | 5 | 2 |  | 3 |  |  | 2 | 2 |  |  |  |  |
| Myrmicaria sani01 |  |  |  |  |  |  |  |  |  |  | 10 |  |  |  |  |  |  |  |  |  |  |  |  |  |  |  |  |  |  |  |  | 39 |
| Paratrechina sani01 |  |  |  |  |  |  |  |  |  |  |  |  |  |  |  |  |  |  |  |  |  |  |  |  | 9 | 4 | 3 | 1 |  | 6 | 11 | 16 |
| Pheidole sani01 |  |  |  |  |  |  |  |  |  |  |  |  |  |  |  |  |  |  |  |  |  |  |  |  |  | 2 | 6 |  | 3 | 10 | 18 | 11 |
| Pheidole sani04 |  |  |  |  |  | 1 |  |  | 5 | 8 | 5 | 5 | 9 |  | 8 | 8 |  | 1 |  |  |  |  |  |  |  |  |  |  |  |  |  |  |
| Pheidole un01 |  |  |  |  |  |  |  |  |  |  |  |  |  | 5 |  | 5 | 4 | 4 | 6 | 4 | 2 | 2 | 4 | 2 |  |  |  | 2 | 1 | 3 | 6 |  |
| Solenopsis sani02 |  |  |  |  | 5 | 2 |  |  | 4 |  |  | 3 |  |  | 4 | 3 | 4 | 1 | 1 | 1 | 4 |  | 7 | 2 | 2 |  |  |  |  | 2 | 4 | 1 |
| Streblognathus peetersi |  |  |  |  |  |  |  |  | 7 | 7 | 5 | 5 | 13 | 4 |  | 9 |  |  |  |  |  |  |  |  |  |  |  |  |  |  |  |  |
| Technomyrmex un01 |  |  |  |  |  |  |  |  |  |  |  |  |  |  |  |  | 4 | 3 | 16 |  | 1 | 25 |  |  |  |  |  |  |  |  |  | 1 |
| Tetramorium sani10 | 1 |  | 1 | 8 | 17 |  | 1 | 19 |  | 3 |  |  |  |  |  |  |  |  |  |  |  |  |  |  |  |  |  |  |  |  |  |  |
| Tetramorium bothae |  |  |  |  |  |  |  |  |  |  |  |  | 2 | 1 | 2 | 5 | 4 |  | 4 | 2 | 4 |  | 3 |  | 3 | 4 | 3 | 3 |  | 4 | 4 | 2 |
| Tetramorium frigidum | 1 |  |  |  |  | 4 | 2 |  | 2 |  | 1 | 3 | 1 |  | 3 | 2 | 6 | 7 | 4 | 2 |  | 3 | 3 |  | 3 |  |  |  |  |  | 4 |  |
| Tetramorium un12 |  |  | 7 | 14 | 3 | 5 | 3 | 3 |  |  |  |  |  |  |  |  |  |  |  |  |  |  |  | 1 |  |  |  |  |  |  |  | 14 |

**Appendix S7: Comparison of our dataset to global ant assemblages**

We used data from Gibb et al. (2017, Ecology 98) on ant assemblages across the globe to put our data in context. This dataset was accessed on 04/02/2019 and contained data on 1423 ant assemblages from across the globe. These assemblages were sourced from 210 studies. We consider each study to comprise a separate species pool for the purpose of our analysis here.


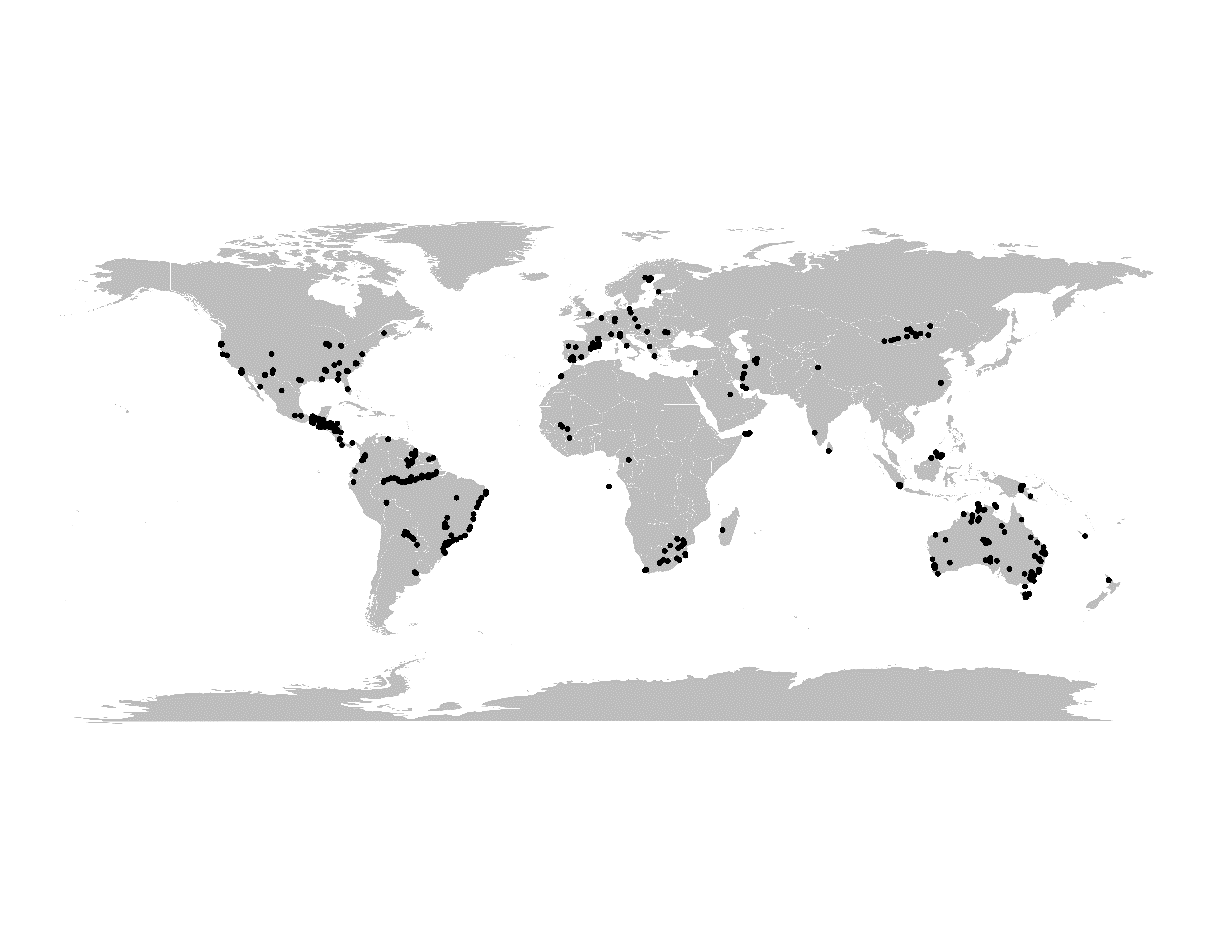
**Figure S7.1.** Distribution of assemblages from the global data set of Gibb et al (2015).

We calculated the total number of species found per study as a measure of species pool size. Summary statistics for the sizes of ant species pools globally are presented in the following table and histogram.

| Table S7.1. Table showing summary statistics for the total number of species found per study in the GlobalAnts database. | | | | | | |
| --- | --- | --- | --- | --- | --- | --- |
| Minimum | 1^st^ Quartile | Median | Mean | 3^rd^ Quartile | Maximum | **This study** |
| 4 | 24 | 50 | 72.08 | 89.75 | 635 | **67** |

**Figure S7.2.** Histogram showing distribution of species pool sizes in Gibb et al (2015). Blue dashed line is size of our measured sample (23), red dashed line is size of the potential species pool that we recorded in 2009 (67).


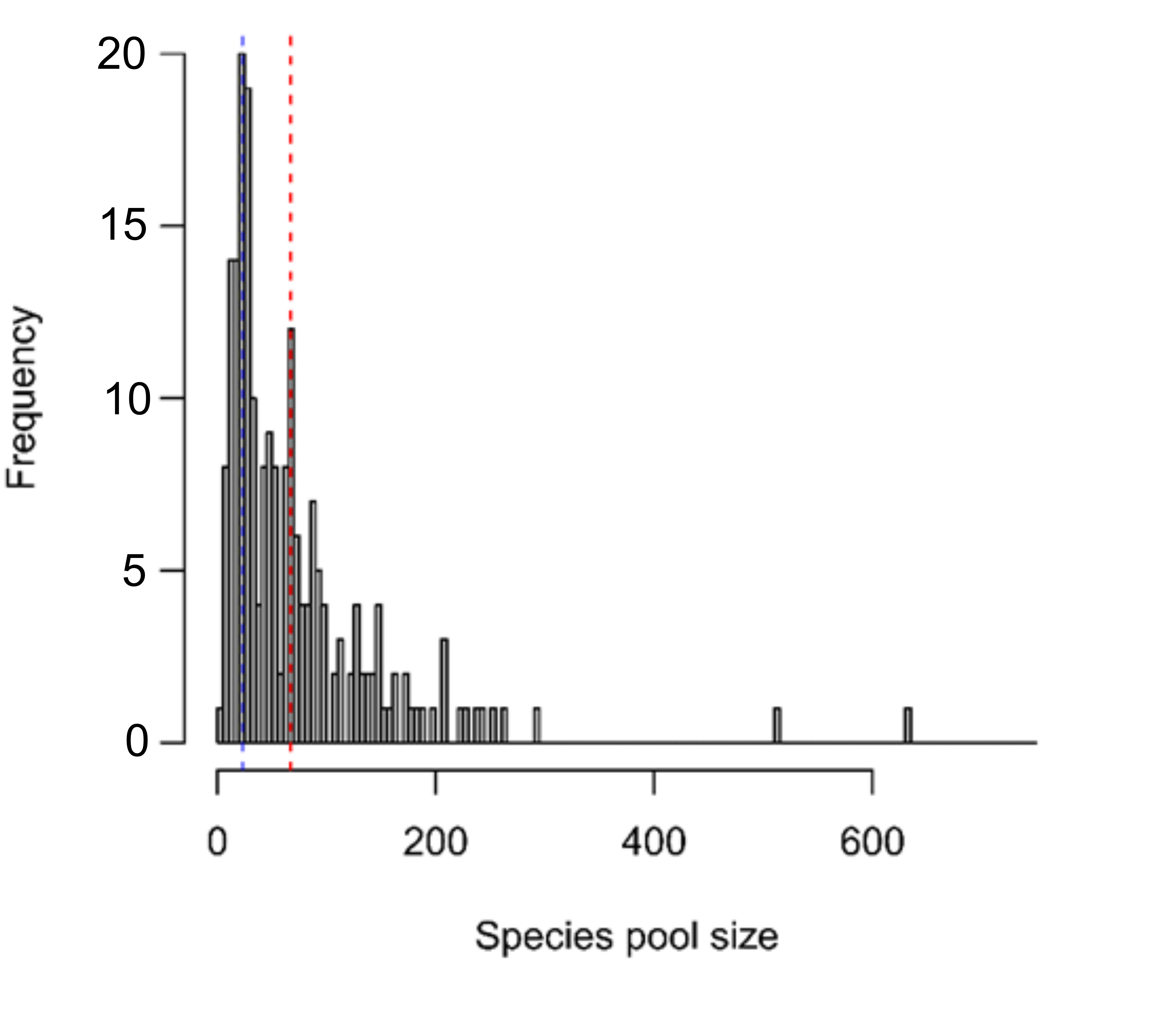


We also calculated a phylogenetic diversity measure, Faith’s index, using a genus level phylogeny (Moreau and Bell 2013, Evolution 67) on each of the 210 studies and compared this to the value for our full species pool (67 species). Our full species pool has above average phylogenetic diversity in comparison to studies from across the globe.

| **Table S7.2.** Table showing summary statistics for Faith’s PD per study in the GlobalAnts database. | | | | | | |
| --- | --- | --- | --- | --- | --- | --- |
| Minimum | 1^st^ Quartile | Median | Mean | 3^rd^ Quartile | Maximum | **This study** |
| 244.8 | 940.1 | 1320.1 | 1365.6 | 1734.9 | 3247.9 | **1594.665** |


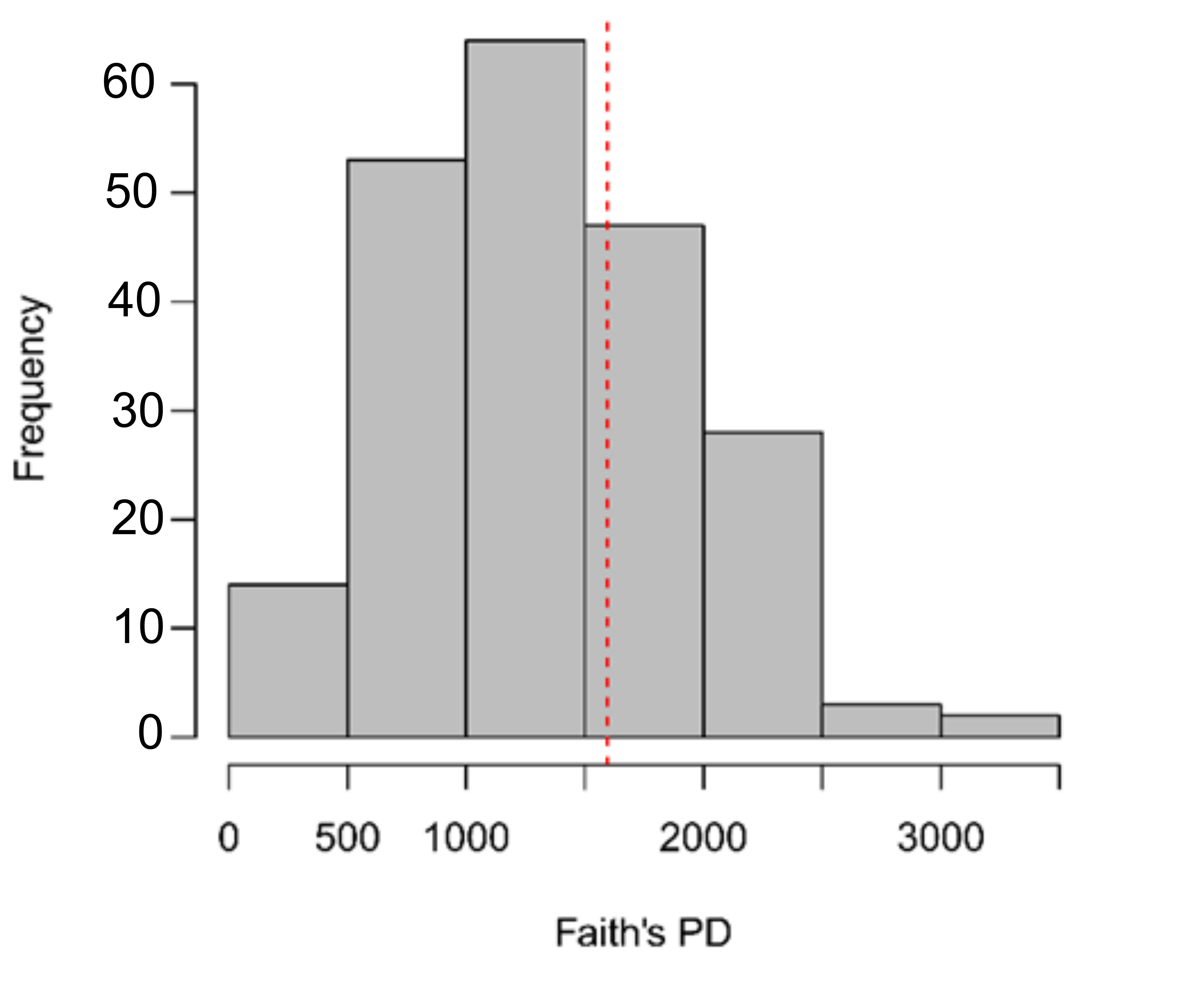


**Figure S7.3.** Histogram showing distribution of phylogenetic diversity in Gibb et al. (2015) studies. Red dashed line is the phylogenetic diversity of the entire species pool (67) we caught in 2009.

These data from GlobalAnts suggest that our wider species pool of 67 is typical of ant species pools across the globe and that our sample of 23 is the same size or larger than 23% of ant species pools globally (48 out of 210 studies had a species pool size of 23 or less). Furthermore, our species pool has above average phylogenetic diversity compared to studies from across the globe.

Through practical necessity, we took a subsample of the possible 67 species we caught in our pitfall traps from the wet season of 2009. First, we only considered species where we had 50 or more individuals available to measure. This was based on the recommendation of Griffiths et al. (2016, Plos One 11). Secondly, we did not have the resources to measure all species that met this threshold. Below is a rank abundance plot showing the species that we sampled in black and the remaining pool in grey. A red dashed line marks the 50 individual threshold:

**Figure S7.4.** Graph showing the rank abundance curve for the ants collected in January 2009 in the Sani Pass. Black dots highlight the subset of 23 species for which we measured traits. The red dashed line marks the 50 individual threshold.


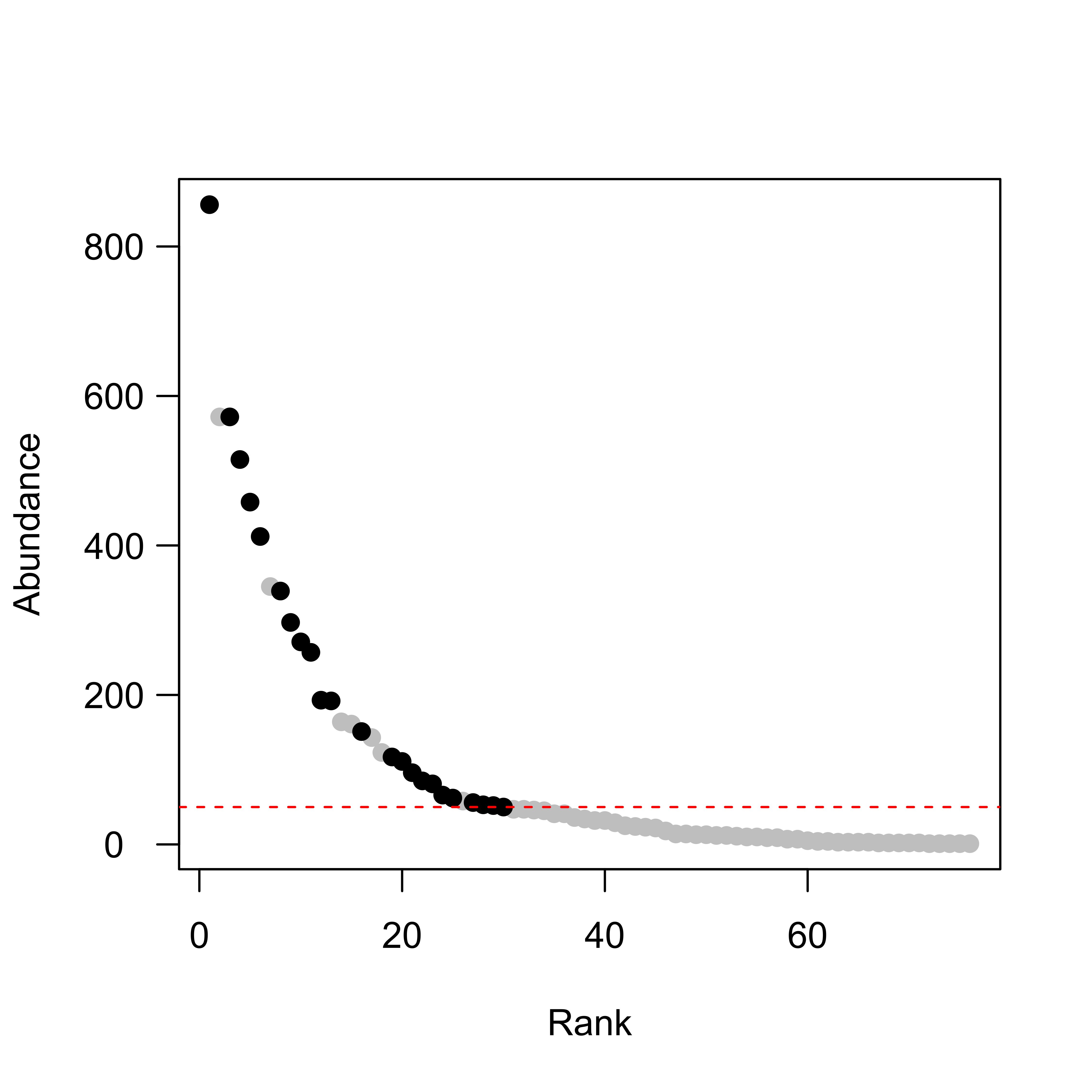


There were 29 species where we caught 50 or more individuals. We sampled 23 of these. The species in our sample represents 70% of the individuals caught in pitfall traps during this season. We sampled 13 genera out of a possible 28. We sampled 4 subfamilies out of a possible 5 in the wider pool (subfamily Dorylinae is not included, only 2 individuals from 2 genera were caught during this season).

Is our particular sample of 23 representative of either the sub-pool of 29 or the entire pool of 67? We test this in terms of phylogenetic and morphological diversity.

We resampled 23 species 100 times each from the two different pools. The first pool was the sub-pool of 29 species for which 50 individuals or more were available. The second was the full species pool of 67. On each of these resamples we calculated a phylogenetic diversity measure, Faith’s index, using a genus level phylogeny (Moreau and Bell 2013, Evolution 67) and functional richness (FRic, Villéger et al. 2008, Ecology 89) using Weber’s length, relative leg length, relative mandible length and relative eye position. Data on these morphological measures for all the species come from our previous publication on this species pool (Bishop et al. 2015, JBI 42).

We ran this resampling analysis to control for species richness. Clearly, our sample has lower phylogenetic and morphological diversity than either the sub-pool or the full pool because it has lower species richness. Thus, the question is whether our sample is representative of the range of possible samples.

The values of Faith’s PD for our sample are average compared to the possible values for the sub-pool or the full pool. The values of FRic for our sample are higher than the average for the sub-pool or the full pool. The following histograms display the range of values we calculated from our resamples on either the sub-pool or the full pool. The dashed red lines indicate the value calculated for our actual sample (the 23 species used in this study).

**Figure S7.5.** Plots showing the distribution of Faith’s PD (upper panels) or FRic (lower panels) calculated from 100 random draws of 23 species from either the sub-pool of species with more than 50 individuals (left panels) or the full species pool (right panels). In each panel, the value for our actual sample of 23 species is shown by the red dashed line.





In sum: (1) our sample represents species comprising 70% of the individuals caught from across the entire gradient; (2) we capture 46% of genera and 80% of subfamilies; (3) we capture an average amount of phylogenetic diversity for a sample of 23 species; (4) we capture an above average amount of morphological diversity for a sample of 23 species.

Given these data and the practical constraints limiting how many species we could reasonably measure, we argue that our sample of 23 species is representative of the wider species pool.

We have shown that the wider species pool on our elevational transect is of typical size and of above average phylogenetic diversity for ant species pools worldwide. We have also shown that our sample of 23 species is also relatively typical in terms of the taxonomic, phylogenetic and morphological make-up of our wider species pool.

Ultimately, we cannot know if these results generalise to larger datasets or, at this stage, any other datasets. Given that our sample appears typical of our wider pool, and our wider pool is actually comparable to others from across the globe, we anticipate that our results are likely to be representative and generalizable.
